# Biological functions, mechanisms, and clinical significance of circular RNA in colorectal cancer

**DOI:** 10.3389/fonc.2023.1138481

**Published:** 2023-03-06

**Authors:** Guida Fang, Dalai Xu, Tao Zhang, Gang Wang, Lei Qiu, Xuzhu Gao, Yongchang Miao

**Affiliations:** ^1^ Department of Gastrointestinal Surgery, Clinical College of Lianyungang Second People’s Hospital, Bengbu Medical College, Lianyungang, China; ^2^ Department of Gastrointestinal Surgery, The Second People’s Hospital of Lianyungang City, Kangda College of Nanjing Medical University, Lianyungang, China; ^3^ Institute of Clinical Oncology, The Second People’s Hospital of Lianyungang City (Cancer Hospital of Lianyungang), Lianyungang, China

**Keywords:** circRNAs, colorectal cancer, biomarker, cancer therapy, cancer diagnosis

## Abstract

Colorectal cancer (CRC) is a leading cause of cancer-related death worldwide due to the lack of effective diagnosis and prognosis biomarkers and therapeutic targets, resulting in poor patient survival rates. Circular RNA (circRNA) is a type of endogenous non-coding RNA (ncRNA) with a closed-loop structure that plays a crucial role in physiological processes and pathological diseases. Recent studies indicate that circRNAs are involved in the diagnosis, prognosis, drug resistance, and development of tumors, particularly in CRC. Therefore, circRNA could be a potential new target for improving CRC diagnosis, prognosis, and treatment. This review focuses on the origin and biological functions of circRNA, summarizes recent research on circRNA’s role in CRC, and discusses the potential use of circRNAs as clinical biomarkers for cancer diagnosis and prognosis, as well as therapeutic targets for CRC treatment.

## 1 Introduction

Colorectal cancer (CRC) is a prevalent malignant tumor worldwide, with the third-highest incidence and the second-highest mortality rate globally (1). The incidence of CRC is increasing worldwide, with over 2.2 million new cases and 1.1 million deaths projected to occur by 2030, according to a recent survey. Developed countries have a higher incidence rate, while developing countries have seen a significant rise in mortality rates (2). The exact mechanism underlying the occurrence and development of CRC is unclear. However, risk factors closely linked to CRC include heredity, diet, smoking, alcohol consumption, physical inactivity, and older age, with age>50 being a particularly significant risk factor. Nonetheless, recent evidence suggests a gradual increase in the incidence of CRC among young people (3). The disease has multifactorial characteristics, and there are no detectable precursors that are universally applicable. Furthermore, there is currently no clinically effective non-invasive screening test to facilitate early diagnosis of CRC (4). There are four subtypes of CRC: CMS1-CMS4, each with different clinical and biological characteristics (5). High-grade CRC tumors are associated with poor prognosis, high metastatic potential, and resistance to conventional therapies, which poses a significant challenge in their treatment. Although our understanding of CRC-related signaling pathways has advanced in recent years, chemotherapy and radiotherapy resistance due to specific molecules in these pathways continues to be a major obstacle to effective treatment (4). Here’s a possible revision: “Non-coding RNAs (ncRNAs) are known to have significant roles in regulating the chemical and radioresistance of CRC, making them promising targets for the development of new anti-drug and anti-radiation therapy strategies (6, 7). These ncRNAs make up the majority (~90%) of the transcribed human genome and include RNAs of varying lengths (8). Numerous studies have demonstrated the involvement of ncRNAs in various physiological and pathological processes, such as diabetes, cardiovascular disease, and cancer (9–11). Short ncRNAs consist of microRNAs (miRNAs), small interfering RNAs (siRNAs), and short piwi-interacting RNAs (piRNAs), whereas both linear lncRNAs (long non-coding RNAs) and circRNAs(Circular RNAs) belong to the category of long non-coding RNAs (12).

CircRNA is a relatively new subtype of ncRNA that was first discovered as a viroid in 1976 (13), and later in eukaryotes (14–16). CircRNA is a single-stranded, covalently closed loop that exhibits strong resistance to exonuclease (17). CircRNA was originally thought to be a non-functional by-product of pre-mRNA splicing (18). However, in recent years, the development and application of high-throughput RNA sequencing (RNA-seq) technology and bioinformatics have identified a large number of circRNAs that play key roles in physiological and developmental processes (19). Moreover, mounting evidence suggests that CircRNA plays a crucial role in tumorigenesis, proliferation, progression, and drug resistance (20, 21). Given its high stability, tissue-specificity, and presence in exosomes and body fluids, circRNA holds great promise as a biomarker or therapeutic target for malignant tumors (21). To elucidate the characteristics and mechanisms of circRNA in CRC, an increasing number of studies are focused on developing new accurate medical biomarkers to identify potential therapeutic targets. Thus, gaining a comprehensive understanding of circRNAs and their roles in CRC signal transduction and molecular mechanisms may aid in identifying more effective treatments. This article aims to introduce the processes and signaling pathways that may be affected by each circRNA during the entire evolution of CRC, with the goal of aiding in early diagnosis, pathological grading, targeted therapy, and prognosis evaluation of CRC.

## 2 The biological origin of circRNA

**Figure 1:**
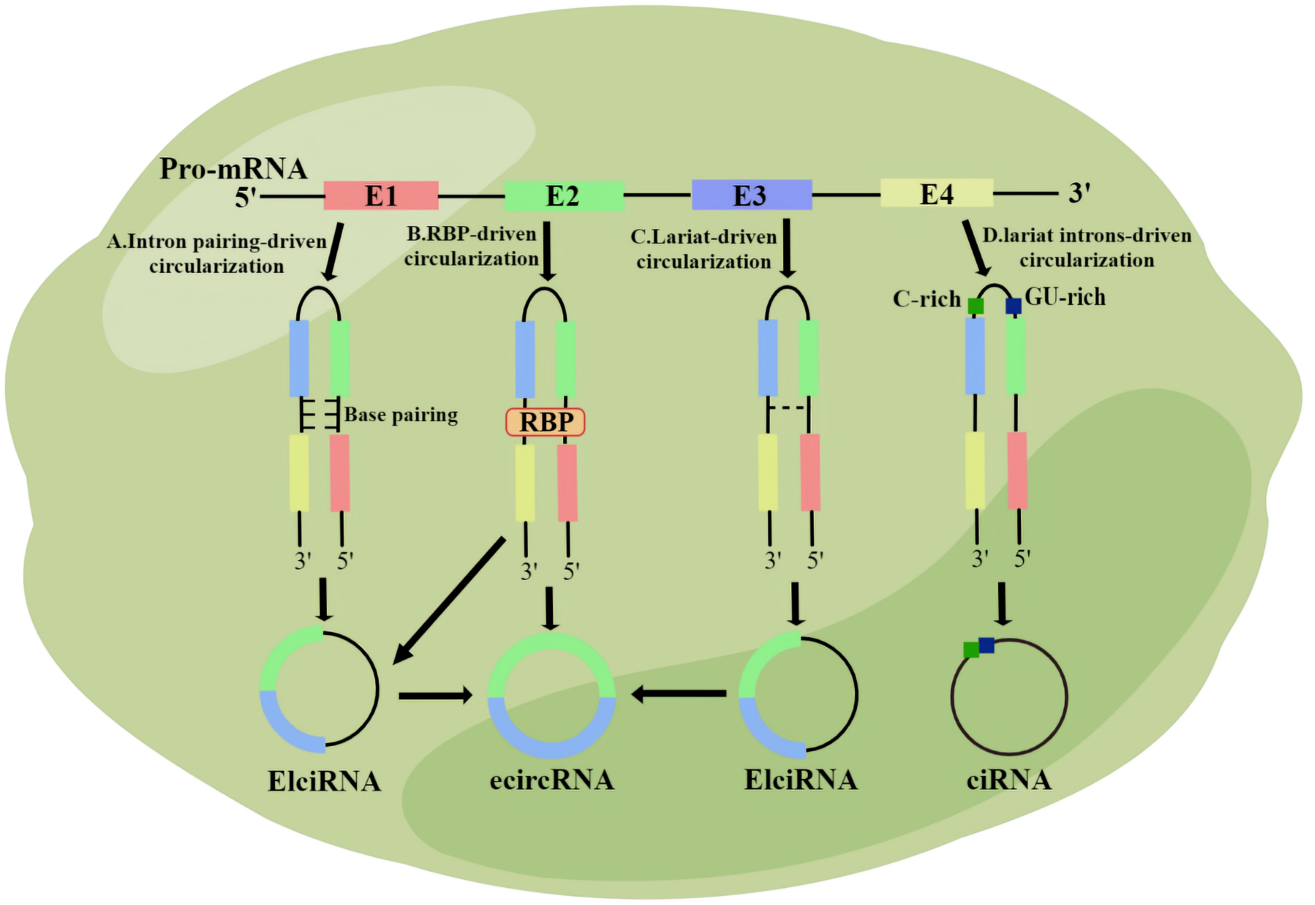
The biological origin of circRNA. **(A)** Intron pairing-driven circularization; **(B)** RNA binding protein (RBP)-driven circularization; **(C)** Lariat-driven circularization; **(D)** Lariat introns-driven circularization.

CircRNA is synthesized during post-transcriptional splicing of pre-mRNA, where mature mRNA is generated after removal of introns by standard splicing. CircRNAs can be classified into four subgroups based on their sequence and domain binding: exonic circRNAs (which are the most common), intronic circRNAs, exonic intron ciRNAs, and tRNA intron ciRNAs (22). Various types of circRNA are produced through different regulation mechanisms. Exon circRNA is produced through both lariat-driven cyclization and reverse splicing (22). In the first pathway, all introns are removed from the lasso, and the remaining exons bind to the 5 ‘ -3 ‘ phosphodiester bond (23).In the second pathway, RNA-binding proteins attached to two introns form eCircRNAs (24, 25). The Alu complementary sequence causes the attachment of two introns during base pairing-driven cyclization (22). Subsequently, reverse splicing leads to the production of EIciRNA (26). Specific circRNAs avoid degradation by linking elements rich in 7 nt GU and elements rich in 11 nt C (27). CiRNA and EIciRNA, located in the nucleus, are important for gene transcription, whereas eCircRNA is widely present in the cytoplasm and does not participate in transcription (22) (**Figure 1**).

## 3 Functions of CircRNA

### 3.1 CircRNA regulates pre-RNA gene splicing and transcription

**Figure 2:**
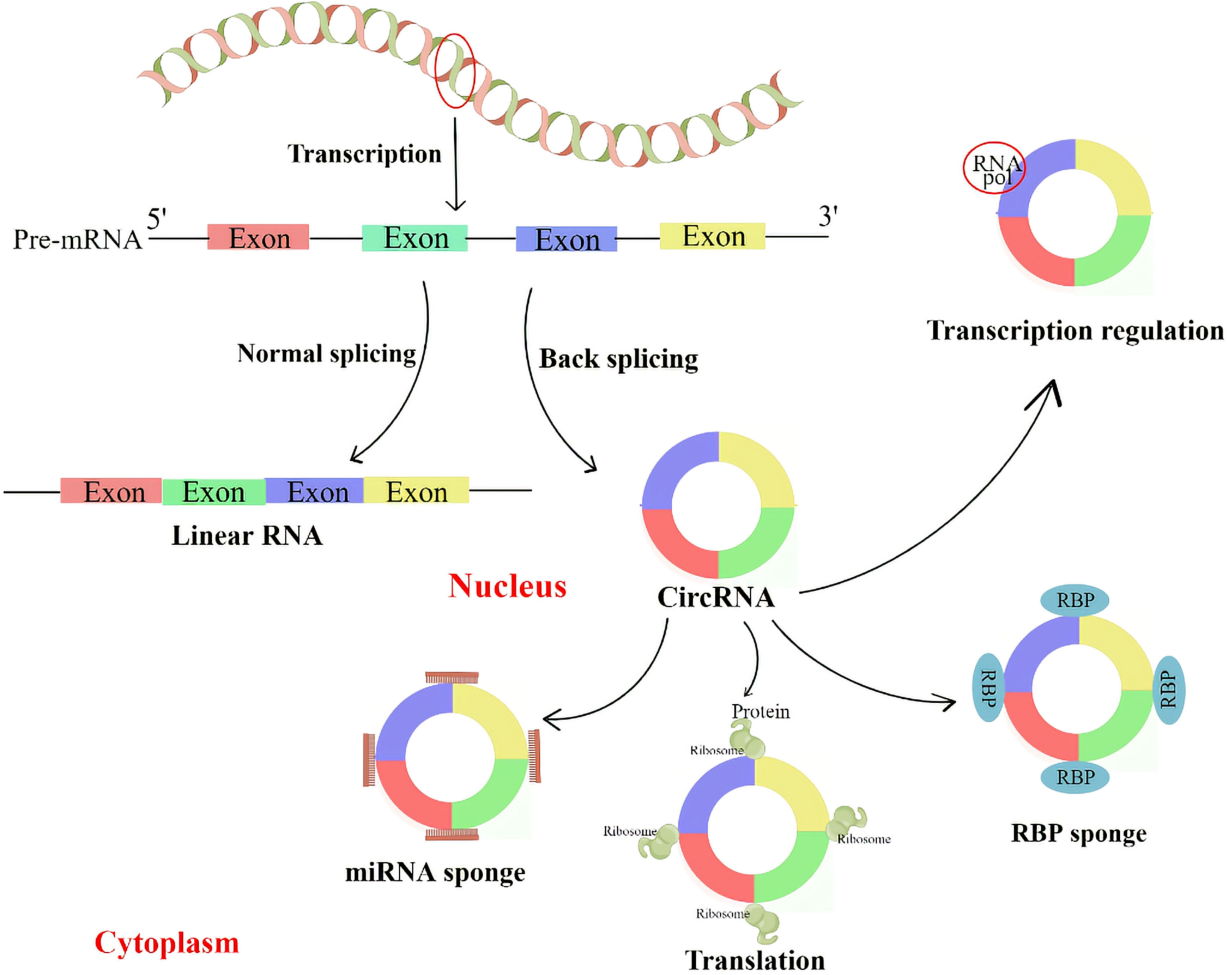
CircRNA regulates the transcription and splicing of parental genes by interacting with RNA polymerase II or transcription-related factors. CircRNA with miRNA binding sites can prevent miRNA from attaching to its target mRNA, thereby blocking the inhibitory effect of miRNA on target proteins. CircRNA can also interact with RNA binding protein (RBP) to regulate protein activity. Some circRNAs can be translated into proteins.

CircRNA can regulate parental genes through competition for splicing sites with linear isoforms. For example, CircMbl, derived from the second exon of the splicing factor muscleblind (MBL), competes with linear MBL mRNA for splicing. Due to the presence of the CircMbl binding site in MBL protein, MBL can tightly bind to CircMbl, thereby promoting CircMbl production. As a result, the expression of CircMbl can be upregulated by MBL expression, leading to a reduction in the production of linear mRNA (28). CircRNA can bind to proteins to regulate the transcription of its locus genes. For example, two eiCircRNAs, Circ-EIF3J and Circ-PAIP2, can form an EIciRNA-U1 complex with U1 small ribonucleoprotein (snRNP), which further targets polymerase II (Pol II) in the promoter region of the host gene and enhances the expression of its parental genes (29). Similarly, ciRNAs such as CircAankrd52 and ci-sirt7 can regulate the transcription of their parental genes through cis-regulation of RNA Pol II (27) (**Figure 2**).

### 3.2 CircRNA as a small RNA sponge

Increasing evidence suggests that some circRNAs are enriched with miRNA response elements (MREs) and can function as miRNAs by competitively binding to other miRNAs. These circRNAs are referred to as competitive endogenous RNAs (ceRNAs) and can sequester miRNAs, thereby relieving their inhibitory effects on target genes (30). MiRNA is another subtype of ncRNA with a length of about 22 nt, and it plays an important role in post-transcriptional regulation by binding to specific sites in the 3’ untranslated regions of mRNA (31, 32). Several lines of evidence have demonstrated that some circRNAs can act as miRNA sponges, including ciRS-7 (33),circSRY (30), circHIPK3 (34), and many other circRNAs (**Figure 2**).

### 3.3 Interaction of CircRNA with RNA-binding proteins

CircRNAs, similar to miRNA sponges, can interact with RNA-binding proteins (RBPs) and act as protein decoys or antagonists. For example, CircRNA derived from Foxo3 (circFoxo3) interacts with cell cycle protein-dependent kinase 2 (CDK2) and cell cycle protein kinase inhibitor 1 (p21), resulting in anticancer and cell cycle blocking effects (35). Other examples, such as CircDNMT1 (36) and circ-NOL10 (37), mainly function as RNA binding protein (RBP) sponges. Recent studies by Okholm et al. (38) have shown that some RBPs preferentially bind to circRNAs rather than their linear counterparts, and this interaction occurs in a cell-type specific manner. They also discovered that circCDYL interacts with IGFBP1 and IGFBP2 in bladder cancer cell lines. Depletion of either circCDYL or these RBPs is indicative of a cancer gene set, and knockdown of this circRNA affects the expression of two important genes involved in tumorigenesis and progression, TP53 and MYC. However, the specific mechanism by which RBPs regulate circRNA requires further investigation (**Figure 2**).

### 3.4 CircRNA is translated into protein

Initially, circRNAs were thought to be ncRNAs because they lack a 5’ cap, a 3’ poly-A tail, and a translation initiation structure (39). However, convincing evidence has shown that some endogenous circRNAs can be translated into proteins. The genome of the hepatitis D virus provides the first example of a circRNA that can be translated into a protein. This single-stranded circRNA encodes the hepatitis D antigen (15). Furthermore, Legnini et al. (40) reported that circZNF609 in mice can be translated into a protein that controls myoblast proliferation *via* the internal ribosome entry site (IRES). Similarly, Pamudurti et al. (41) found that circMbl can be translated into proteins in the head of flies in a cap-independent manner. Additionally, circAKT3 and circFBXW7 (42) were found to encode proteins that inhibit tumorigenesis in human gliomas **Figure 2**).

## 4 The expression of circRNA in CRC

Thus far, a cumulative sum of 173 circRNAs have been identified *via* PubMed and documented as differential expressions in the tumorous and adjacent normal tissues of CRC. Amongst these, 67 circRNAs exhibited an up-regulation, while 23 demonstrated a down-regulation in both cell-culture experiments and animal studies. **Tables 1** and **2** depict the prominence of up-regulated circRNAs over their down-regulated counterparts. Throughout the evaluation of these 90 circRNAs, their regulatory impact on fundamental malignant traits has been comprehensively illustrated, encompassing factors such as proliferation, migration, invasion, and apoptosis.

**Table 1:** Up-regulated circRNA in CRC.

CircRNA	GENE Related miRNA	Expression	Targeted molecules/pathways	Function	References	
Circ_0011385	miR-330-3p	up	MYO6	Proliferation, migration and invasion	(43)	2022
Circ_0000231	miR-375	up	CCND2	Proliferation as well as tumorigenesis	(44)	2022
Circ_0001955	miR-583	up	FGF21	Facilitate CRC cell malignancy, promote tumor growth	(45)	2022
Circ_0003602	miR-149-5p	up	SLC38A1	Migration and invasion	(46)	2022
Circ_0052184	miR-604	up	HOXA9	Proliferation, migration, and invasion	(47)	2022
Circ_0006732	miR-127-5p	up	RAB3D	Proliferation, migration, invasion and EMT of CRC cells	(48)	2022
Circ_0056618	miR-411-5p	up	PRRG4	Proliferation, migration, invasion and EMT of CRC cells	(49)	2022
Circ_0082182	miR-326	up	NFIB	OXA resistance, proliferation, invasion,migration, suppress apoptosis	(50)	2022
Circ_0001535	miR-485-5p	up	LASP1	Proliferation, invasion, stemness, and tumor growth	(51)	2022
Circ_0044556	miR-665	up	Diaphanous Homolog 1	Proliferation, migration, invasion and EMT of CRC	(52)	2022
Circ_0006174	miR-1205	up	CCBE1/Wnt	Promote cell growth,metastatic and suppress cell apoptosis	(53)	2022
Circ_0060927	miR-421 and miR-195-5p	up	Caudatin	Proliferation, migration and invasion, and induced cell apoptosis	(54)	2022
Circ_0045932	miR-873-5p	up	HK2	Proliferation, invasion, migration, and glycolysis abilities	(55)	2022
Circ_0081069	miR-665	up	E2F3	Proliferation, migration and invasion	(56)	2022
Circ-CD44	miR-330-5p	up	ABCC1	OXA-resistant, resistance, proliferation, migration, and invasion, suppress apoptosis	(57)	2022
Circ_0001550	miR-4262	up	NUCKS1	Proliferation, metastasis, stemness, and hinder apoptosis.	(58)	2022
Circ_0004585	miR-874-3p	up	CCND1	5-FU resistance,colony formation,migration,invasion, and inhibit apoptosis	(59)	2022
Circ_0000523	miR-let-7b	up	METTL3	Inhibit cell viability and promote apoptosis and invasion	(60)	2022
Circ_0007334	miR-577	up	KLF12	Migration, invasion, and angiogenesis	(61)	2022
Circ_KIAA1199	miR-34c-5p	up	MSI1	Proliferation, survival, migration and invasion	(62)	2022
Circ-FAT1	miR-619-5p	up	FOSL2	Migration, invasion, and angiogenesis	(63)	2022
Circ_0007031	miR-485-3p	up	MELK	Proliferation,repress apoptosis	(64)	2022
Circ_0006174	miR-1205	up	CCND2	Promote DOX resistant	(65)	2022
Circ_0014130	miR-197-3p	up	PFKFB3	5-FU resistance, promote proliferation, inhibit apoptosis	(66)	2022
Circ_0067717	miR-497-5p	up	SLC7A5	Proliferation, invasion, glutamine metabolism, and Restrain apoptosis	(67)	2022
Circ_0068464	miR-383	up	Wnt/β-catenin	Migration proliferation and inhibit apoptosis	(68)	2022
Circ_0030998	miR-567	up	VEGFA	Proliferation,angiogenesis	(69)	2021
Circ_0089153	miR-198	up	SENP1	Proliferation, sphere formation ability, and repress cell apoptosis	(70)	2021
Circ_0000467	miR-382-5p	up	EN2	Multiplication, migration, invasion, EMT	(71)	2021
Circ-RNF121	miR-1224-5p	up	FOXM1	Proliferation, migration, invasion and glycolysis repress apoptosis	(72)	2021
Circ_0062682	miR-940	up	PHGDH	Promotes serine metabolism and tumor growth	(73)	2021
Circ_0007142	miR-874-3p	up	GDPD5	Proliferation,reduce apoptosis and ferroptosis	(74)	2021
Circ-ACAP2	miR-143-3p	up	FZD4	Proliferation, migration, invasion and radioresistance,inhibit the apoptosis	(75)	2021
Circ_0136666	miR-497	up	PD-L1	Proliferation,reduce apoptosis	(76)	2021
Circ-MFN2	miR-574-3p	up	IGF1R	Proliferation, migration, invasion, and radioresistance	(77)	2021
Circ-KRT6C	miR-485-3p	up	PDL1	Growth, migration, invasion, and immune escape, suppress apoptosis	(78)	2021
Circ_0040809	miR-515-5p	up	DNMT1	Proliferation, migration, invasion, reduce apoptosis	(79)	2021
Circ-SIRT1		up	EIF4A3/N-cadherin	Proliferation, invasion, and EMT	(80)	2021
Circ_0006174	miR-138-5p	up	MACC1	Migration proliferation and inhibit apoptosis	(81)	2021
Circ_0101802	miR-1236-3p	up	MACC1	Proliferation, migration, invasion	(82)	2021
Circ-0000212	miR-491	up	FOXP4	Proliferation	(83)	2021
Circ_0000338	miR-217 and miR-485-3p	up		5-FU resistance	(84)	2021
Circ_0084615	miR-599	up	DNMT3A	Proliferation, migration, invasion	(85)	2021
Circ_0029803	miR-216b-5p	up	SKIL	Proliferation, migration, invasion, EMT and glycolysis	(86)	2021
Circ_0026416	miR-545-3p	up	MYO6	Proliferation, migration, invasion, EMT	(87)	2021
Circ_DOCK1	miR-132-3p	up	USP11	Migration proliferation and inhibit apoptosis	(88)	2021
Circ_0031787		up	Wnt/β-catenin	Proliferation and invasion, increase tumor growth	(89)	2021
Circ_0071589	miR-526b-3p	up	KLF12	CDDP resistance, proliferation, migration and invasion, and inhibit apoptosis	(90)	2021
Circ_0087862	miR-142-3p	up	BACH1	Proliferation, migration, invasion	(91)	2021
Circ-Erbin	miR-125a-5p and miR-138-5p	up	4EBP-1	Proliferation, migration and metastasis	(92)	2020
Circ_0053277	miR-2467-3p	up	MMP14	Proliferation, migration, and EMT	(93)	2020
Circ-0004277	miR-512-5p	up	PTMA	Proliferation, inhibit apoptosis	(94)	2020
Circ_0026416	miR-346	up	NFIB	Proliferation, migration and invasion	(95)	2020
Circ_0128846	miR-1184	up	AJUBA	Increase tumor growth, proliferation	(96)	2020
Circ_0001178	miR-382/587/616	up	ZEB1	Migratory and invasive	(97)	2020
Circ_0001806	miR-193-5p	up	COL1A1	Increasing sphere-formation ability	(98)	2020
Circ_0000512	miR-296-5p	up	RUNX1	Proliferation, inhibit apoptosis	(99)	2020
Circ_0032833	miR-125-5p	up	MSI1	5-FU and OXA resistant	(100)	2020
Circ_0007142	miR-122-5p	up	CDC25A	Proliferation, colony formation, migration, and invasion	(101)	2020
Circ_0005576	miR-874	up	CDK8	Proliferation, inhibit apoptosis	(102)	2020
Circ-FARSA	miR-330-5p	up	LASP1	Proliferation, migration and invasion	(103)	2020
Circ_0007031	miR-133b	up	ABCC5	5-FU resistance, cell colony formation and invasion	(104)	2020
Circ_0056618	miR-206	up	CXCR4 and VEGF-A	Proliferation, migration and angiogenesis	(105)	2020
Circ_0136666	miR-383	up	CREB1	Promote the proliferation and glycolysis and inhibit the apoptosis	(106)	2020
Circ-PRKDC	miR-198	up	DDR1	5-FU resistance, cell colony formation and invasion	(107, 108)	2020
Circ_0137008	mi-338-5p	up	GRIK3	Proliferation, migration	(109)	2020
Circ-101555	miR-597-5p	up	CDK6 and RPA3	Proliferation, inhibit apoptosis	(110)	2019
Circ_0136666	miR-136	up	SH2B1	Proliferation, migration, invasion	(111)	2019
Circ_0007142	miR-103a-2-5p	up		Proliferation, migration, and invasion	(112)	2019
Circ-0104631		up	PTEN/Akt/mTOR	Increase tumor growth, migration	(113)	2019
Circ_0005075		up	Wnt/β-catenin	Proliferation, migration, and invasion	(114)	2019
Circ_0079993	miR-203a-3p.1	up	CREB1	Proliferation	(115)	2019
Circ_0071589	miR-600	up	EZH2	tumor growth, invasion and migration	(116)	2018
Cirs7	miR−7	up	EGFR and IGF1R	Promotes progression	(117)	2017
Circ_000984	miR-106b	up	CDK6	Promotes cells proliferation and metastasis	(118)	2017
Circ_0020397	miR-138	up	TERT and PD-L1	Proliferation and invasion	(119)	2017

**Table 2:** Down-regulated circRNA in CRC.

CircRNA	GENE Related miRNA	Expression	Targeted molecules/pathways	Function	References	
Circ-LECRC	miR-135b-5p	down	KLF4	Inhibit CRC cell proliferation,migration, and invasion and promoted apoptosis	(120)	2022
Circ_0003215	miR-663	down	DLG4	Inhibit cell proliferation,migration, invasion,and CRC tumor metastasis	(121)	2022
Circ_0000826		down	AUF1	Inhibit cell proliferation	(122)	2022
Circ_0094343	miR-766-5p	down	TRIM67	Inhibit proliferation, clone formation, glycolysis, and 5-FU,L-OHP,Dox resistance	(123)	2022
Circ_0065378	miR-4701-5p	down	TUSC1	Inhibit cell proliferation, cell invasion, migration, and EMT	(124)	2022
Circ_0007919	miR-942-5p	down	TET1	suppress CRC cell growth and migration	(125)	2022
Circ_0003266	miR-503-5p	down	PDCD4	repressed CRC cell proliferation, migration, and invasion, and accelerated the cell apoptosis	(126)	2021
Circ_0005927	miR-942-5p	down	BATF2	repress cell colony-forming ability, migration and invasion,induce cell apoptosis	(127)	2021
Circ_0021977	miR-10b-5p	down	p21 and p53	suppress proliferation, migration, and invasion	(128)	2020
Circ_0137008	miR-338-5p	down		suppressed the migration, invasion, and EMT	(129)	2020
Circ-SMARCA5	miR-39-3p	down	ARID4B	Inhibit CRC cell proliferation, migration and invasion	(130)	2020
Circ_0008285	miR-382-5p	down	PTEN/PI3K/AKT	Inhibit proliferation and migration	(131)	2020
Circ-NOL10	miR−135a−5p	down	KLF9	Inhibit proliferation, cell cycle, migration, and invasion	(132)	2020
Circ-TADA2A	miR−374a−3p	down	KLF14	Inhibit Tumor suppressor in CRC	(133)	2020
Circ-CSNK1G1	miR−455−3p	down	MYO6	Inhibit Proliferation, migration and invasion cell growth and metastasis,	(134)	2020
Circ_0007142	miR−122−5p	down	CDC25A	Inhibit Proliferation, colony formation, migration, and invasion	(135)	2020
Circ_0009361	miR-582	down	APC2	Inhibit proliferation, EMT, migration, and invasion	(136)	2019
Circ_0026344	miR-183	down	CCL20 and CXCL8	Inhibit Proliferation, migration and invasion cell growth	(137)	2019
Circ-ITGA7	miR-3187-3p	down	ASXL1	Inhibit Proliferation	(138)	2019
Circ-CDYL7	miR−150−5p	down	c−Myc cyclin D1	Inhibits CRC cell growth and migration	(139)	2019
Circ-0014717		down	P16	Inhibits CRC cell growth	(140)	2018
Circ_0026344	miR-21 and miR-31	down		decreased the growth and invasion, promoting apoptosis	(141)	2018
Circ_0000523		down	Wnt/β-catenin	decreased the growth, promoting apoptosis	(142)	2018
Circ-ITCH	miR−7, miR−17, miR−214	down	DDX17 WNT/β−catenin	Inhibit Proliferation	(143)	2015

### 4.1 Up-regulated circRNA in CRC

Wang et al. (43) have recently verified that the upregulation of circ_0011385 and MYO6 in both CRC tissues and cells was significant, while the down-regulation of miR-330-3p was observed. Notably, the expression of circ_0011385 in CRC patients exhibited a positive correlation with tumor size and TNM stage. Furthermore, the inhibition of cell proliferation, migration, and invasion and the induction of apoptosis in CRC cells were observed upon silencing of circ_0011385 or down-regulation of MYO6. Remarkably, the inhibitory effect of circ_0011385 silencing on CRC progression was reversed by the down-regulation of miR-330-3p or the overexpression of MYO6. It is noteworthy that circ_0011385 is interrelated with miR-330-3p, and the latter targets MYO6. The *in vivo* overexpression of Circ_0011385 leads to the promotion of tumor growth through the miR-330-3p/MYO6 axis.

The up-regulation of circ_0006732 has been observed in the tissues of CRC. Further investigation has revealed that circ_0006732 serves as a competitive endogenous RNA (ceRNA), which directly interacts with miR-127-3p, thereby affecting the expression of Ras-related protein Rab-3D (Rab3D). Notably, it has been verified that the down-regulation of circ_0006732 can significantly suppress the proliferation, migration, invasion, and EMT of CRC cells by regulating the miR-127-5p/RAB3D axis. Based on these findings, Circ_0006732 emerges as a prospective therapeutic target for the treatment of CRC (48).

Circ_0056618 and PRRG4 were found to be up-regulated in both CRC tumor tissues and cells, while miR-411-5p exhibited down-regulation. The suppression of CRC cell proliferation, migration, invasion, and EMT was observed following the knockdown of Circ_0056618 or PRRG4. The regulation of PRRG4 expression was positively influenced by Circ_0056618 through the targeting of miR-411-5p. Furthermore, the inhibition of CRC cell proliferation, migration, invasion, and EMT, which were induced by the knockdown of circ_0056618, were rescued by miR-411-5p deletion or PRRG4 overexpression. Animal studies have confirmed the hindrance of tumor growth *in vivo* due to circ_0056618 knockdown. Therefore, the targeting of the circ_0056618/miR-411-5p/PRRG4 axis could be a promising therapeutic approach for treating CRC (49).

Zou Y et al. (68) reported an abnormal up-regulation of circ_0068464 in both CRC cells and tissues when compared to their normal counterparts. The knockdown of circ_0068464 inhibited the migration and proliferation of CRC cells, induced apoptosis, and decreased the expression of Wnt/β-catenin pathway-related proteins such as β-catenin, cyclin D1, C-myc, and LEF-1. *In vivo* tumorigenicity experiments in nude mice further confirmed the inhibitory effect of circ_0068464 down-regulation on tumor growth and lung metastasis. Additionally, the interaction between circ_0068464 and miR-383 was established through dual luciferase assay and RNA immunoprecipitation assay. It was found that miR-383 was significantly downregulated in CRC tissues and cells, and the inhibition of miR-383 expression reversed the inhibitory effect of circ_0068464 knockout on CRC cells. Thus, it was concluded that circ_0068464 promotes CRC development by targeting miR-383 to regulate the activation of the Wnt/β-catenin pathway.

### 4.2 Down-regulated circRNA in CRC

In a recent study, Chen B et al. (121) reported the down-regulation of circ_0003215 in CRC, which was negatively correlated with tumor size, TNM stage, and lymph node metastasis. The authors found that the decrease in circ_0003215 level was due to the RNA degradation by m6A read-write protein YTHDF2. Functional assays revealed that Circ_0003215 inhibited cell proliferation, migration, invasion, and metastasis both *in vitro* and *in vivo*. The study also demonstrated that Circ_0003215 regulates the expression of DLG4 through miR-663b sponge, thereby inducing metabolic reprogramming in CRC. Mechanistically, DLG4 inhibits the pentose phosphate pathway (PPP) by K48-linked ubiquitination of glucose-6-phosphate dehydrogenase (G6PD).

In CRC tissues and cell lines, a significant downregulation of circ_0007919 and TET1 gene expression was observed, while miR-942-5p exhibited an upregulated expression. Remarkably, *in vitro* studies demonstrated that the overexpression of circ_0007919 led to a substantial inhibition of both CRC cell growth and migration. Moreover, we discovered that circ_0007919 acts as a competitive inhibitor, binding to miR-942-5p, thereby increasing the expression of the downstream target gene TET1. Altogether, our results reveal that circ_0007919 plays a crucial role in suppressing the oncogenic behavior of CRC cells, functioning through the modulation of the miR-942-5p/TET1 axis. These findings offer a novel therapeutic avenue for the management of CRC (125).

Yan D S et al. (124) have reported a significant reduction in the expression of the circRNA, circ_0065378, in CRC tissues. *In vitro* experiments demonstrated that the upregulation of circ_0065378 impeded the malignant behavior of CRC cells, as evidenced by the suppression of cellular proliferation, invasion, migration, and epithelial-mesenchymal transition (EMT). At the molecular level, we observed that circ_0065378-mediated tumor suppression was exerted through the sequestration of miR-4701-5p. Remarkably, we found that the knockdown of the TUSC1 gene expression, a direct downstream target of miR-4701-5p, partially abolished the inhibitory effects of either miR-4701-5p inhibitor or circ_0065378 overexpression on the malignant behavior of CRC cells. These findings collectively suggest that the circ_0065378/miR-4701-5p/TUSC1 axis constitutes a promising molecular target for the diagnosis and treatment of CRC.

## 5 CircRNAs as potential therapeutic targets

Given the emerging regulatory role of circRNAs in cancer, their potential as effective therapeutic targets is gaining momentum. However, it has been reported that resistance to certain circRNAs can occur during the treatment of CRC (**Table 3**).

**Table 3:** CircRNAs upregulated or downregulated in CRC, with the implication in therapy resistance.

CicrRNA	miRNA	Targeted molecules/pathways	Biological Function	Expression	References
Circ_0006174	miR-1205	CCND2, wnt/β-catenin	DOX resistance, migration, and invasion	up	(65)
Circ_0014130	miR-197-3p	PFKFB3	5-FU resistance, promote proliferation, Inhibit apoptosis	up	(66)
Circ_0082182	miR-326	NFIB, AKT pathway	OXA resistance, proliferation, invasion,migration, suppress apoptosis	up	(50)
Circ_0094343	miR-766-5p	TRIM67	Inhibit 5-FU,OXA,Dox resistance,proliferation, clone formation, glycolysis	down	(123)
Circ_0004585	miR-874-3p	CCND1	5-FU resistance,colony formation,migration,invasion, and Inhibit apoptosis	up	(59)
Circ-CD44	miR-330-5p	ABCC1	OXA-resistant, resistance, proliferation, migration, and invasion, suppress apoptosis	up	(57)
Circ_0000338	miR-217 and miR-485-3p		5-FU resistance,Inhibit apoptosis	up	(84)
Circ_0071589	miR-526b-3p	KLF12	CDDP resistance, proliferation, migration and invasion, and Inhibit apoptosis	up	(90)
Circ-PRKDC	miR-375	FOXM1, wnt/β-catenin	5-FU resistance, cell colony formation and invasion	up	(108)
Circ_0007031	miR-133b	ABCC5,BCL2,AKT pathway	5-FU resistance, cell colony formation and invasion	up	(104)
Circ_0032833	miR-125-5p	MSI1	5-FU and OXA resistant,proliferation and invasion	up	(100)

Circ_0006174 exhibits upregulation in doxorubicin (Dox)-resistant CRC cells and tissues. Notably, the depletion of Circ_0006174 instigated a reduction in Dox resistance, cell proliferation, migration, and invasion of CRC cells. Remarkably, the copious expression of circ_0006174 has been found to emanate from exosomes of Dox-resistant CRC cells. Additionally, Zhang et al. established the targeting association between circ_0006174/miR-1205 or miR-1205/CCND2. The exosomal Circ_0006174 enforces Dox resistance by upregulating CCND2 *via* miR-1205 mediation. *In vivo*, circ_0006174 knockdown also enhances tumor sensitivity to Dox by targeting the miR-1205/CCND2 axis. Taken together, these findings signify that exosomes enriched in Circ_0006174 could be utilized as a diagnostic biomarker for chemoresistance in CRC (65).

A mounting body of evidence indicates that exosomes originating from drug-resistant cells can engender resistance in chemosensitive cells. The exosome’s structure contributes to the prevention of RNA degradation, which guarantees an effective concentration of circRNA. The size and membrane structure of exosomes also promote the absorption and fusion of cancer cells. The expression of Circ_0094343 was significantly reduced in CRC tissues, chemoresistant CRC tissues, and metastatic CRC tissues. Furthermore, the exosome-borne Circ_0094343 also impedes HCT116 cell proliferation, clone formation, and glycolysis. Notably, Circ_0094343 augments the chemosensitivity of HCT116 cells to 5-fluorouracil (5-FU), oxaliplatin (L-OHP), and doxorubicin (Dox). Moreover, Circ_0094343 functions as a sponge for miR-766-5p, targeting and regulating TRIM67. The results demonstrate that Circ_0094343 hinders the proliferation, clone formation, and glycolysis of HCT116 cells through the miR-766-5p/TRIM67 axis, thereby instigating chemoresistance (123).

Wang Z F et al. have discovered that circ_0082182 is upregulated in OXA-resistant CRC tissues and cells. Circ_0082182 downregulation impedes drug resistance, proliferation, invasion, and migration of OXA, while promoting apoptosis in OXA-resistant CRC cells. MiR-326 directly targets NFIB, preventing OXA resistance in CRC cells and thwarting cancer development. Circ_0082182 regulates NFIB expression by acting as a sponge for miR-326. In OXA-resistant xenograft tumor models, Circ_0082182 mediates the miR-326/NFIB axis, promoting tumor growth. Consequently, Circ_0082182 boosts NFIB expression by sequestering miR-326, thereby regulating the onset and progression of OXA resistance and CRC. Therefore, Circ_0082182 could be a prospective therapeutic target for OXA-resistant CRC (50).

**Figure 3:**
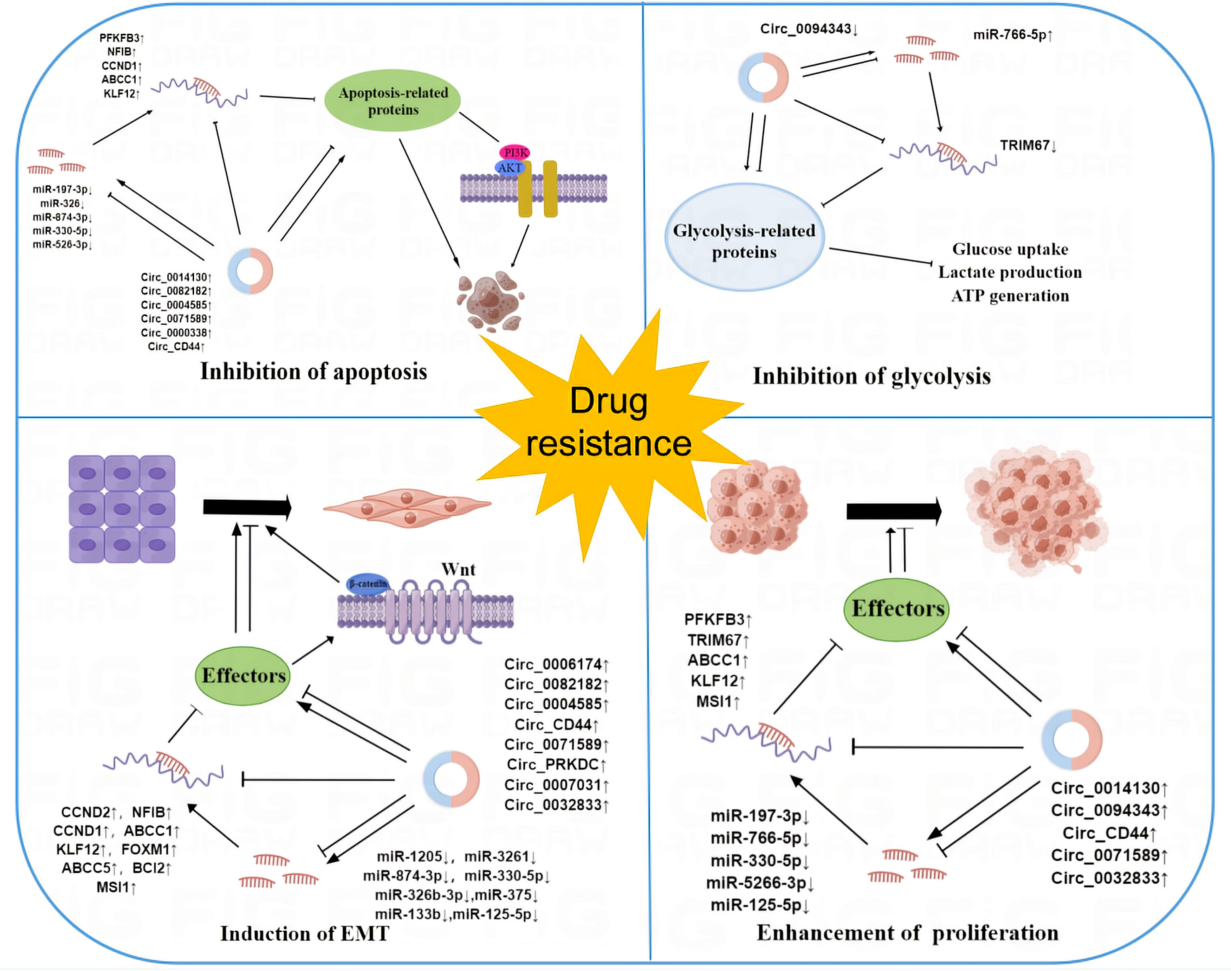
The mechanism of circRNAs in cancer drug resistance. Dysregulation of circRNAs contributes to the development of cancer resistance by regulating a variety of cellular processes in cancer cells, such as apoptosis, EMT, glycolysis, and cell proliferation.

These findings provide novel insights into our comprehension of the mechanisms behind circRNA-mediated resistance. Their dysregulation propels the evolution of drug resistance in cancer through various mechanisms, including apoptosis inhibition, EMT induction, cell proliferation promotion, and glycolysis suppression (**Figure 3**). However, the precise mechanism remains incompletely understood. As our comprehension of circRNA deepens, circRNA may hold great clinical promise in the future.

## 6 CircRNA as a diagnostic biomarker for CRC

**Figure 4:**
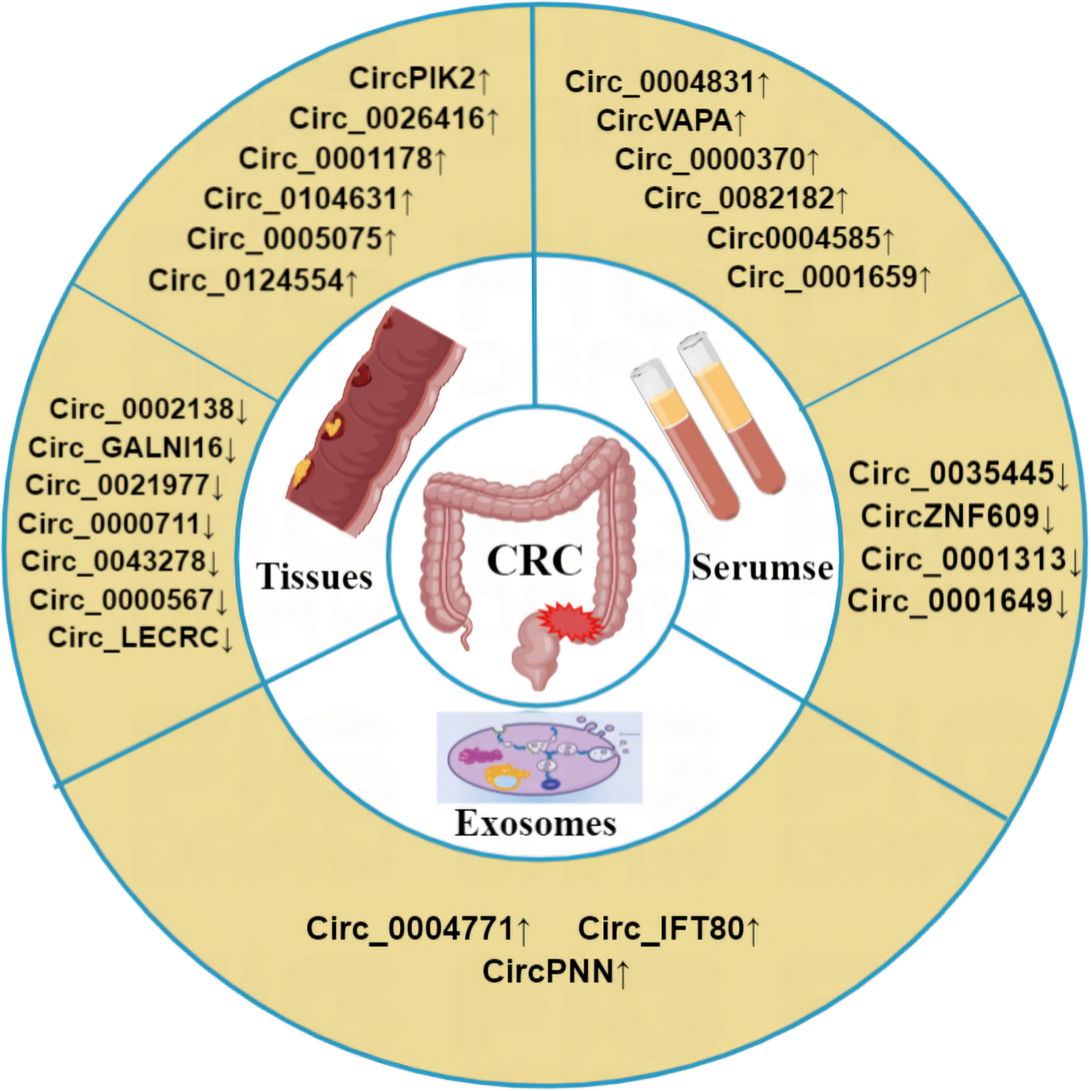
Abnormal expression of circRNA in CRC. Compared with the normal control group, circRNA was differentially expressed in CRC tissues, exosomes and blood of CRC patients. ‘↑’ means up, ‘↓’ means down.

Due to the high mortality rate associated with CRC, there is an urgent need for early diagnostic and prognostic indicators. As circRNAs are abundantly expressed in cancer cells, they represent a promising class of biomarkers for CRC. Indeed, clinical samples, including CRC patient tissues and plasma, have demonstrated the potential of circRNAs as valuable biomarkers for early diagnosis and metastasis prediction of CRC (**Table 4**, **Figure 4**).

**Table 4:** Clinical significances of dysregulated circRNAs in CRC.

CircRNA	Expression	Sample	Clinicopathological Association	Potential Function	AUC	Sensitivity/Specificity (%)	References
Circ_0002138	down	tissues	differentiation grade	Prognosis/ Diagnosis	0.724		(144)
Circ-GALNT16	down	tissues	tumor size, tumor stage, and lymph node metastasis	Prognosis/ Diagnosis			(145)
CircPTK2	up	tissues	distxlal metastases	Prognosis			(146)
Circ_0021977	down	tissues	TNM stage	Prognosis/ Diagnosis			(128)
Circ_0026416	up	tissues	pTNM, stage, tumour differentiation	Prognosis	0.767		(95)
Circ_0004831	up	serums	differentiation grade	Diagnosis			(147)
CircVAPA	up	serums	lymphovascular invasion,TNM stage	Diagnosis	0.724		(148)
Circ_0000370	up	serums	lymph node metastasis	Diagnosis	0.815		(149)
Circ_0082182	up	serums	lymph node metastasis	Diagnosis	0.737		(149)
Circ_0035445	down	serums	TNM stage	Diagnosis	0.702		(149)
CircZNF609	down	serums	tumor diameter	Diagnosis	0.767		(150)
Circ_0001313	down	serums	tumor size, differentiation grade	Diagnosis			(151)
Circ_0001649	down	serums	pathological differentiation	Diagnosis	0.857		(152)
Circ-0004771	up	exosomes	TNM stage	Prognosis	0.59		(153)
CircIFT80	up	exosomes	tumor stage, distant metastasis, and tumor size	Prognosis			(154)
CircPNN	up	exosomes	TNM stage, tumor size, lymph node metastasis	Prognosis	0.554		(155)
Circ_0001178	up	tissues	TNM stage	Diagnosis			(97)
Circ-0104631	up	tissues	TNM stage	Diagnosis			(113)
Circ_0005075	up	tissues	histology/differentiation, depth of invasion,TNM stage	Diagnosis			(114)
Circ_0000711	down	tissues	Differentiation, TNM stage	Prognosis/Diagnosis	0.81	0.91,0.58	(156)
Circ_0004585	up	tissues, serums	LNM	Prognosis	0.5		(157)
Circ_0043278	down	tissues	tumor differentiation stage,LNM	Prognosis	0.71	0.72,0.70	(158)
Circ_0001659	up	tissues, serums	Differentiation grade	Prognosis/ Diagnosis	0.87	0.67, 0.91	(159)
Circ_0006282	up	serums	TMN stage, LNM	Prognosis	0.831	0.788,0.769	(160)
Circ_0000567	down	tissues	TMN stage, LNM, Differentiation grade, depth of invasion	Prognosis	0.865	0.833,0.764	(161)
Circ-LECRC	down	tissues	TNM stage	Prognosis			(120)
Circ_0124554	up	tissues	LNLM1 or LNLM0	Prognosis/ Diagnosis			(162)

A recent study by Mohammadi et al. has demonstrated that the expression level of circ_0006282 is significantly increased in CRC tissues and plasma samples of CRC patients when compared to healthy controls (p<0.0001). The area under the curve (AUC) was 0.831 (95%CI:0.779-0.883), suggesting that circ_0006282 has the potential to serve as a biomarker for the early detection and metastasis prediction of CRC. Importantly, the expression of circ_0006282 in CRC patients decreased to normal levels after surgery (p<0.0001), indicating its potential role as a monitoring tool. When combined with carcinoembryonic antigens (CEA) and carbohydrate antigen 199 (CA199), the use of circ_0006282 showed high specificity and sensitivity in CRC detection. Furthermore, the study revealed that plasma circ_0006282 can differentiate between patients with colorectal cancer and colitis. Taken together, these findings suggest that plasma circ_0006282 can serve as a promising diagnostic and dynamic monitoring biomarker for CRC (160).

Exosomes represent a critical modulator of intercellular communication and have been the subject of numerous scientific inquiries. These vesicles, originated from cancerous cells, are able to encapsulate circRNAs and release them into the bloodstream (163). Furthermore, there has been a surge of interest in using exosome-derived circRNAs as disease biomarkers (164). Notably, Pan B et al. have observed a marked upregulation of exosomal circ_0004771 in the serum of CRC patients. The use of GW4869, a known inhibitor of exosome secretion, led to a significant reduction in the level of exosomal circ_0004771 in the medium, without causing a noteworthy change in CRC cells. Thus, the presence of circulating exosomal circ_0004771 is a promising candidate as a diagnostic biomarker for early detection of CRC (153).

## 7 Conclusion and prospect

As a nascent variety of ncRNA, CircRNA participates in diverse physiological processes and pathological conditions. While the precise role of circRNAs is still a matter of debate, mounting evidence supports their function as miRNA sponges, protein decoys, or translational modulators, as well as their role in regulating pre-RNA gene splicing and transcription. Presently, CircRNA represents a frontier in tumor biology and treatment, with no less than hundreds of aberrantly expressed circRNAs detected in CRC tissues, many of which have been demonstrated to modulate CRC cell proliferation, migration, invasion, and apoptosis. These circRNAs have the potential to serve as diagnostic biomarkers and effective therapeutic targets for CRC. Despite novel perspectives on the clinical application of circRNA, experimental and clinical research in CRC lags behind. The molecular mechanisms underlying the cyclization, degradation, and intracellular localization of circRNAs in CRC remain elusive. Furthermore, the biological functions and mechanisms of the majority of circRNAs in CRC are still poorly understood, and alternative functions or mechanisms beyond miRNA sponges are yet to be uncovered. Despite the diagnostic and prognostic potential of certain circRNAs, their clinical utility is limited by low sensitivity and specificity. Therefore, it is crucial to investigate the differential expression of circRNAs in clinically relevant samples, such as blood and urine, in addition to CRC tissues, and to improve their sensitivity and specificity. Liquid biopsy, owing to its minimal or non-invasive nature and widespread acceptance, holds great promise as a potent tool for early screening, diagnosis, and prognosis of cancer. Furthermore, the ability to collect liquid biopsy samples at predetermined intervals facilitates monitoring of cancer treatment efficacy, drug resistance, as well as recurrence and metastasis.

This review comprehensively summarizes the current research progress of circRNA in the diagnosis, prognosis, progression, and drug resistance of CRC, highlighting its immense potential as a novel biomarker for early diagnosis, prognosis, and treatment. However, despite the significant strides that have been made, there is still much to be learned about the regulatory mechanism of circRNAs, and their downstream regulatory networks and clinical correlations. An in-depth understanding of these aspects will undoubtedly enhance our understanding of the role of circRNAs in CRC and pave the way for the development of circRNA-based diagnosis, prognosis, and treatment strategies for CRC. As biological methods and information technology continue to advance, it is expected that an increasing number of circRNAs associated with CRC and their physiological and pathological functions will be discovered. Although the precise mechanisms of circularization, degradation, cell localization, and function of circRNAs in CRC remain largely unknown, the enigma of circRNA will eventually be unraveled. New diagnostic and therapeutic strategies based on circRNAs will undoubtedly find their way into clinical practice in the future.

## Author contributions

Study concept and design: GF and YM. Analysis and interpretation of data: GF, DX and TZ. Drafting of the manuscript: GF and DX. Critical revision of the manuscript for important intellectual content: GF, DX, GW, LQ, XG and YM. Obtained funding: GF and YM. Study supervision: TZ and YM. All authors contributed to the article and approved the submitted version.
